# Supplementary material for: Low incidence of HCC in chronic hepatitis C patients with pretreatment liver stiffness measurements below 17.5 kilopascal who achieve SVR following DAAs
Source: PLoS One. 2020 Dec 10;15(12):e0243725. doi: 10.1371/journal.pone.0243725 (PMC7728240; doi:10.1371/journal.pone.0243725)
Supplement: S1 Appendix — (DOCX) [file pone.0243725.s001.docx]

*Heavy alcohol use:*

Registration of alcohol treatment in *the National Registry of Alcohol Treatment (NRAT)*, registration of a prescription with ATC-code N07BB01 - N07BB05 in *the* *Danish National Prescription Registry* or a diagnosis in the *Danish National Patient Register* of ICD-10 codes: K 70.0 – 70.9, F 10.2 – 10.9, G 32.1, G 62.1, G 72.1, I 42.6, K 86.0, T 50.0A.

*HIV infection*

Registration of a diagnosis of HIV infection in DANHEP or in *The Danish National Patient Register* of ICD-10 codes: B 20.0 – B 24.9

*Hepatitis B*

Registration of chronic hepatitis B in DANHEP and at least one of the following: a positive test for HBs-antigen, HBV DBA or HBV genotype.

*Injection drug use*

Registration of intravenous drug use in DANHEP or registration of intravenous use of opioids (heroin, methadone, morphine or buprenorphine) in *the Registry of Drug Abusers Undergoing Treatment.*

*Decompensated cirrhosis*

Registration of a complication to cirrhosis (ascites, variceal bleeding, hepatic coma, or spontaneous bacterial peritonitis) in DANHEP or a diagnosis in *the Danish National Patient Register* of ICD-10 codes: K 70.3 – 70.4, K 71.7, K72.1, K 74.3-74.4, K 74.6-74.9, K 76.6 – 76.7, K 65.8I, R 18.9.

*Hepatocellular carcinoma*

Registration in *the Danish Pathology Register* of SNOMED codes: *M81703-M81707* or a diagnosis in the *Danish National Patient Register* of ICD-10 codes: C 22.0. A registration in the *Danish Cancer Registry* of hepatocellular carcinoma.

*Diabetes*

Registration of a diagnosis of diabetes in DANHEP or a diagnosis in *the Danish National Patient Register* of ICD-10 codes: E10.0 – 14.9.

*Western European origin*

Registration in DANHEP of Western European origin
