# Supplementary material for: Low incidence of HCC in chronic hepatitis C patients with pretreatment liver stiffness measurements below 17.5 kilopascal who achieve SVR following DAAs
Source: PLoS One. 2020 Dec 10;15(12):e0243725. doi: 10.1371/journal.pone.0243725 (PMC7728240; doi:10.1371/journal.pone.0243725)
Supplement: Attachment — Submitted filename: Response to Reviewers .pdf [file pone.0243725.s002.pdf]

Response to Reviewers

We would like to thank the academic editor and the reviewers for their comments.

We have addressed the queries in order below

Response to the academic editor

1: Editor's comment: Please ensure that your manuscript meets PLOS ONE's style requirements, including those for file naming.

Author's reply: We have tried to adhere to the PLOS ONE's style requirements.

2: Editor's comment: We note that you have indicated that data from this study are available upon request. PLOS only allows data to be available upon request if there are legal or ethical restrictions on sharing data publicly.

Author's reply: We have addressed the issue of data access in the cover letter.

Response to reviewer 1:

1: reviewer's comment: „We did not find a significantly higher risk of developing HCC in patients with a pLSM of 10-17.4 kPa compared to patients with a pLSM <10 kPa".
LSM is influenced by several factors, e.g. it has been shown that values can be higher in patients that have had a meal withing the last 2 hours prior to the investigation. This needs to be considered. In addition, pretreatment LSM can be influenced by the ALT value (inflammation). Ideally, a cut-off at the end of the treatment could be more accurate? (i.e. Clin Infect Dis. 2019 Nov 22;ciz1140.)

Authors's reply: We have discussed the first point in the discussion:
Thirdly, we did not have data on whether pLSM were performed with the patients fasting. As LSM can be falsely elevated if the patient is not fasting this could have caused an overestimation of the LSM cutoffs in the study.
However, patients in Denmark are instructed to be fasting when having LSM performed and as almost all patients had at least one LSM prior to the pLSM, most would be expected to have been fasting at the time of the pLSM.
We also agree that also having a LSM at end of treatment or SVR would have been preferable, but unfortunately, it was not available in most patients. We have added the following to the discussion: It would have been preferable to also have LSM at end of treatment (EOT) or at SVR as the inflammation caused by CHC can cause an elevation of LSM, regardless of fibrosis and LSM at SVR could be more accurate at predicting outcomes after DAA treatment [38]. However, LSM at EOT or SVR was not available in most patients. Furthermore, a significant part of patients in treatment for CHC are lost to follow up, especially vulnerable patients like those with active injecting drug use or suffering from homelessness [39]. Being able to provide prognostication and reassurance at the time of treatment initiation would be important, especially in patients at risk of lost to follow up after treatment.

2: reviewer's comment: "Only 11 patients in that cohort developed HCC. 9/11 patients could be identified by the cut-off of 17.5 kPa. The authors mentioned that LSM >17.5 kPA was associated with age, male sex and diabetes. All these factors have been associated with HCC risk. Were the other 2 patients male, had older age and diabetes? Are these factors (in combination) more relevant than LSM? I wonder if the authors would not perform HCC surveillance in a male patient, with LSM of 14 kPA and diabetes mellitus? Thus, in my view the concluison is too stroing in my view based on the 11 patients with HCC."

Author's reply:
We have described the two patients with a LSM <17.4 under the heading "Hepatocellular carcinoma": Of the two patients who were diagnosed with HCC post treatment, the first was a female in her mid-fifties with a pLSM of 4.7 kPa and no history of heavy alcohol use or diabetes while the other was a male in his late fifties with a pLSM of 13.0 kPa and a history of both heavy alcohol use and diabetes.
We also added the following sentence in the discussion:
"As the negative predictive value for the cut-off of 17.5 kPa was very high at 99.7 %, this could suggest that within the first three years after cure for hepatitis C, monoinfected patients with no prior episode of HCC or decompensated cirrhosis and with a pretreatment LSM below 17.5 kPa may not benefit from HCC surveillance. However, patients with a pLSM of 10-17.4 kPa and other risk factors for HCC should also be considered for HCC screening, based on individual risk assessment."

3: reviewer's comment: LSM may not be available in all settings. Other easier to use parameter may be more valuable.
Several studies have investigated albumin (J Hepatol. 2020 Mar;72(3):472-480) or FIB-4 (e.g. Gastroenterology. 2019 Nov;157(5):1264-1278.e4.) as predictive marker. FIB-4 data could improve the study.

Author's reply: We agree that adding biomarkers would have improved the study. Unfortunately,blood samples, including albumin and especially ASAT were only available for a minority of patients in the study and we therefore could not include albumin or FIB-4 in the analyses.
We have mentioned this in discussion: It would have been a great advantage to the study if serological markers of liver fibrosis, such as FIB-4, had been available to corroborate our LSM findings, but as aspartate aminotransferase (AST) was not a standard test in Denmark during the study period, and only available for a small proportion of patients.
We also agree that the use of LSM is not available in all settings, limiting the applicability of the study, but when available, it can be practical in outreach programs among the most vulnerable patients where one contact prior to treatment initiation (using LSM and POC HCV RNA testing) is preferable.

We have added the following text in the introduction: Using LSM as a predicting marker allows for less contacts with health care providers before treatment initiation, as compared to biomarkers. This can be advantageous in outreach programs among marginalized populations, such as homeless people and people who inject drugs.

<u>Response to reviewer 2:</u>

1: reviewer's comment: A valid LSM <2 years before treatment with DAA which is the index day might be quite long and might include a selection bias – subgroup analysis or time between LSM and index date as confounder in the multivariate would be important to show.

Author's reply: We agree that the long time from LSM to index date in some patients could represent a bias and have included the time between LSM and index date as a variable in the regression analyses.

2: reviewer's comment: the primary outcome was HCC but more patient died than developing HCC during follow, authors should state and discuss how they dealt with competing risk (death before HCC). The figures show cumulative incidences which suggest that this was considered during analysis.

Author's reply: We agree that competing risk analysis should be used for HCC and decompensated cirrhosis and have changed from cox regression to competing risk regression and reported subhazard ratios for these outcomes.

3: reviewer's comment: Multivariate data might be over fitted as incidence of 11 (HCC), 14 (dec. cirrhosis) and 38 for death restrict the number of confounder to maximum of four when calculating the risk of death and even 1-2 for the other endpoints.

Authors reply: We have lowered the cutoff for including variables in multivariate analyzes from $p<0.1$ to $p<0.05$, thus lowering the number of confounders included.
