# Supplementary material for: Low incidence of HCC in chronic hepatitis C patients with pretreatment liver stiffness measurements below 17.5 kilopascal who achieve SVR following DAAs
Source: PLoS One. 2020 Dec 10;15(12):e0243725. doi: 10.1371/journal.pone.0243725 (PMC7728240; doi:10.1371/journal.pone.0243725)
Supplement: Attachment — Submitted filename: Response to Reviewers.docx [file pone.0243725.s003.docx]

**Response to Reviewers**

We would like to thank the reviewers for their comments.

We have addressed the queries in order below

**Response to reviewer 2:**

**1: reviewer’s comment:** Authors should mention in the stats section or results section why this analysis was necessary - as death was competing substantially with the occurrence of HCC or complications of cirrhosis.

**Author’s reply:** We agree that this should be specified and have rewritten the section in the statistics section:

Cox regression was used to estimate hazard ratios for all-cause death. Competing risk regression was used to estimate subhazard ratios for HCC and decompensated cirrhosis, as death was a substantial competing risk for these two outcomes.
